# *DOK7*, a target of miR-299-5p, suppresses the progression of bladder cancer

**DOI:** 10.18632/aging.205304

**Published:** 2023-12-13

**Authors:** Xuemei Tian, Dan Liu, Peng He, Lijun Li, Yu Wang, Mingxing Qiu

**Affiliations:** 1 Department of Anesthesia Surgery Center, Sichuan Provincial People’s Hospital, School of Medicine, University of Electronic Science and Technology of China, Chengdu 610072, China; 2 Department of Urology, Sichuan Provincial People’s Hospital, School of Medicine, University of Electronic Science and Technology of China, Chengdu 610072, China

**Keywords:** *DOK7*, BLCA, miR-299-5p, JAK signaling pathway

## Abstract

Objective: Bladder cancer (BLCA) is the 6th most common malignancy in males. microRNA (miRNAs) can function as tumor suppressors or oncogenic factors, which are of significance in the progression of BLCA. This study explored the mechanisms by which miR-299-5p modulates *DOK7* (Docking Protein 7) expression and the functional role of *DOK7* in the progression of BLCA.

Methods: The expression of the *DOK7* in BLCA patient samples was examined by RT-qPCR (Real-time quantitative polymerase chain reaction), Western blotting and Immunohistochemical (IHC) staining. The malignant phenotype of BLCA cells upon *DOK7* overexpression or silencing was assessed by functional assays including cell count kit-9 (CCK8), colony formation and 5-ethynyl-2’-deoxyuridine (Edu) staining assays, as well as Transwell migration and invasion assays. The miRNA regulators of *DOK7* were identified through bioinformatics prediction, and the biological role of miR-299-5p/*DOK7* axis was validated by functional assays. The impact of miR-299-5p/*DOK7* axis on Janus kinase (JAK)/signal transducer and activator of transcription (STAT) pathway was further examined by Western blotting.

Results: *DOX7* was significantly downregulated in BLCA tumor tissues compared with normal tissues. Ectopic *DOK7* expression suppressed the proliferation, migration and invasion of BLCA cells. *DOK7* overexpression also attenuated the tumorigenesis of BLCA cells in nude mice. miR-299-5p was a negative regulator of *DOK7* expression in BLCA cells. miR-299-5p/*DOK7* axis impaired the malignancy of BLCA cells through regulating the JAK signaling pathway.

Conclusion: Our data indicate that miR-299-5p/*DOK7* axis suppresses BLCA progression possibly by regulating the JAK signaling pathway.

## INTRODUCTION

Bladder cancer (BLCA) is one of the common malignancies in the urinary system, with increasing incidence worldwide in recent years [1]. There is a lack of the understanding of the molecular mechanisms underlying the occurrence and progression of BLCA. Apart from surgical resection, there are limited therapeutic options for the effective treatment for BLCA patients at present [2–6]. Accumulating evidence suggests that genetic, epigenetic and environmental factors are involved in the tumorigenesis and development of BLCA [7–10]. Understanding the molecular mechanisms related to the progression of BLCA tumors is of great significance for the development of new therapeutic targets.

microRNAs (miRNAs) are a class of short non-coding RNAs which function to modulate different cellular processes related to cancer progression, including cell proliferation, migration, epithelial-mesenchymal transition, and drug resistance [11, 12]. miRNAs can act as tumor suppressor or oncogenic factors by targeting the expression of oncogene and tumor-suppressor genes [13]. For example, miR-99a was found as a tumor suppressor factor in BLCA, and its down-regulation induced the malignant phenotype in BLCA cells [14]. miR-154 was identified as another tumor suppressor whose low expression was correlated with the dismal prognosis in BLCA patients, and miR-154 overexpression attenuated the aggressive phenotype of BLCA cells [15]. In contrast, miR-96 was found to be an oncogenic miRNA whose overexpression promotes the progression of BLCA [16]. Therefore, miRNAs could serve as novel prognostic biomarker and therapeutic targets in BLCA.

*DOK7* (Docking Protein 7) has been characterized as gene involved in postsynaptic differentiation and the neural activation of muscle-specific receptor kinases [17]. The mutation of *DOK7* is one of the major causes of congenital myasthenic syndrome [18]. Emerging evidence suggests that *DOK7* functions as a tumor suppressor in different cancers. For instance, *DOK7* was found to be down-regulated in glioma and its down-regulation facilitates the growth of glioma cells [19]. In breast cancer, *DOK7* overexpression could suppress the cell proliferation and mobility by dampening phosphoinositide 3-kinase (PI3K)/Protein kinase B (AKT) signaling pathway [20]. Nevertheless, there is currently no reports regarding the functional role of *DOK7* in BLCA.

Janus kinase (JAK)-signal transducer and activator of transcription (STAT) signaling pathway was initially characterized as an important regulator in immune cell activation [21]. Apart from acting as a regulator in immune surveillance, the aberrant activation of JAK-STAT signaling has been recognized as a key driver in tumor progression [22]. On the other hand, JAK-STAT signaling inhibition could suppress the malignant phenotype in BLCA cells. For example, Rac family of small GTPase 3 (RAC3) was found to enhance the aggressiveness of BLCA cells by activating JAK-STAT Signaling [23]. ST8 alpha-N-acetyl-neuraminide alpha-2,8-sialyltransferase 1 (ST8SIA1) was reported to impair the malignancy of BLCA cells by blocking the JAK/STAT signaling pathway [24]. Direct inhibition of STAT3/5 activity also hinders the cell growth of BLCA cells [25]. However, the mechanism by which JAK-STAT signaling become deregulated in BLCA has not been fully understood.

Previous reports demonstrated that miR-299-5p is abnormally expressed in different tumors [26, 27], while its expression pattern in BLCA tumors and cell lines has not been studied. Here, we attempted to investigate the functional role of *DOK7* and the potential upstream miRNA regulator in BLCA. The bioinformatics analysis and *in vitro* experiments demonstrated the functional interaction between miR-299-5p and *DOK7* in BLCA cells. We further investigated the role of miR-299-5p/*DOK7* axis in dictating the aggressive phenotype of BLCA cells and regulating the tumorigenesis in the animal model.

## MATERIALS AND METHODS

### Clinical sample collection

In this study, we collected BLCA specimens and the matched para-cancerous samples by surgery from 108 BLCA patients diagnosed at Sichuan Academy of Medical Sciences and Sichuan Provincial People’s Hospital. Inclusion criteria: patients diagnosed with primary BLCA without medical record of chronic diseases. Exclusion criteria: patients who were diagnosed with other type of cancers; patients who had undergone preoperative radiotherapy, chemotherapy or hormone drug administration. All the clinical samples were stored in −80°C freezer. All the subjects provided the informed written letter of consent. The patients were followed for more than 30 months to measure the overall survival. The study was approved by Medical Research Ethics Committee of Sichuan Academy of Medical Sciences and Sichuan Provincial People’s Hospital (No. 2019-139).

### Bioinformatics analysis

The prediction of miRNAs targeting *DOK7* were predicted using the *DOK7* mRNA sequence based on the following databases: “Starbase” (http://starbase.sysu.edu.cn), “Targetscan” (http://www.targetscan.org/ miRanda) and “miRDB” (https://mirdb.org/). The survival of BLCA patients and *DOK7* expression level data were retrieved from Gene Expression Profiling Interactive Analysis (GEPIA) online tool (http://gepia.cancer-pku.cn/) based on BLCA dataset from The Cancer Genome Atlas (TCGA) database ((https://tcga-data.nci.nih.gov/tcga). Gene set enrichment analysis was conducted using GSEA software [28].

### Cell culture and the generation of stable *DOX7* expression or knockdown cell lines

Human BLCA cell lines (RT4, T24, UMUC3, and 5637), human normal bladder cell line (SV-HU-1), and human embryonic kidney cell line (HEK293) were maintained with DMEM medium (GE™ Hyclone, UT, USA) containing 10% FBS (GE™ Hyclone, UT, USA), 100 U/ml penicillin and 100 mg/ml streptomycin (Thermo Fisher Scientific Co. Ltd., MA, USA) in a humidified atmosphere of 37°C and 5% CO2.

We constructed T24 and RT4 cells stably expressing *DOK7* or shRNA targeting *DOX7* using the lentiviral system. A combination of pSPAX2, pMD2.g and PCDH-*DOK7*-puro or PCDH-sh-*DOK7* plasmids (GenePharma, Shanghai, China) were co-transfected into HEK293 cells using Lipofectamine 3000 (Thermo Fisher Scientific Co. Ltd., MA, USA). The empty vector of PCDH-puro or control shRNA vector PCDH-sh-Ctrl was used to produce the control lentivirus for *DOK7* overexpression or *DOK7* knockdown. 48 hours after transfection, the virus supernatant was collected to transduce T24 and RT4 cells in the presence of polybrene (8 μg/mL, Beyotime, Beijing, China). The infected cells were selected with 0.5 μg/ml puromycin (Beyotime, Beijing, China) for 7 days to eliminated uninfected cells. The following experimental groups were set up: vector (cells transduced with lentivirus carrying empty vector of PCDH-puro, control group for overexpression); *DOK7* (cells transduced with lentivirus carrying PCDH-*DOK7*-puro, *DOK7* overexpression group); sh-NC (cells transduced with lentivirus carrying control shRNA, control group for *DOK7* knockdown); sh-*DOK7* (cells transduced with lentivirus carrying *DOK7* shRNA, *DOK7* knockdown group).

### Animals and xenograft model

All animal work protocols were approved by Sichuan Academy of Medical Sciences and Sichuan Provincial People’s Hospital Committee for Animal Care and Use (No. 20220816002). Twelve 6–8-week-old (weighed 25–30 g) BALB/C nude mice purchased from Beijing Vital River Laboratory Animal Technology Co., Ltd. (Beijing, China) were housed in the standard SPF animal facility with 60–65% humidity and a 12-hour light/dark cycle at 22–25°C. The mice were fed with AIN-76 purified rodent diet (Daici Biotech, Wuci, China), and the mice were allowed with food and drinking water ad libitum. After the acclimation for 7 days, the mice were randomly assigned into two experimental groups (*n* = 6 mice in each group): the vector group (subcutaneously injected with 1 × 10^6^ T24 cells stably expressing the control empty vector after lentivirus infection) and *DOK7* group (injected with 1 × 10^6^ T24 cells stably expressing *DOK7* vector after lentivirus infection). The tumor volume was monitored every week post-injection using a caliper and the volume was determined using the formula: V(tumor) = 0.5 × length × width^2^ (mm^3^). The success of tumor formation was validated after 1 or 2 weeks by the observation of the subcutaneous xenograft formation. 35 days after the subcutaneous injection of tumor cells all the mice were euthanized by cervical dislocation [15]. The xenograft tumor samples were collected for subsequent analyses.

### Real-time quantitative PCR (RT-qPCR)

Total RNA from cell and tissue samples were extracted using Trizol reagent (Beyotime Biotechnology, Beijing, China), and afterward the RNA samples were reverse transcribed into complementary DNA (cDNA) using BeyoRT™ II First Strand cDNA Synthesis Kit (Beyotime Biotechnology, Shanghai, China). The TB Green^®^ Fast qPCR Mix (Takara Biomedical Technology (Beijing) Co., Ltd., Beijing, China) Kit was used to perform the real-time qPCR analysis. The PCR cycling condition used: 95°C 2 min for initial denaturation, 40 cycles of 95°C 30 sec denaturation, 60°C 30 sec annealing and 72°C 60 sec extension, with signal detection at the end of each cycle. Finally, the 2^−ΔΔCt^ method was used to analyze the relative expression level and glyceraldehyde-3-phosphatedehydrogenase (*GAPDH*) was used as the internal reference gene. The primer sequences used in the experiments are shown below:

*DOK7*-Forward: 5′-GCCATCATGCTGGGCTTTGACA-3′
*DOK7*-Reverse: 5′-AACTTGGTGCCTGGAGCCACTG-3′
*GAPDH*-Forward: 5′-GTCTCCTCTGACTTCAACAGCG-3′
*GAPDH*-Reverse: 5′-ACCACCCTGTTGCTGTAGCCAA-3′

### Western blot

Total protein was extracted from tissues and cells using RIPA lysis buffer containing protease inhibitor cocktail (Thermo Fisher Scientific, MA, USA). The protein concentration in the cell lysate was measured by a BCA Protein Assay Kit (Zeye Biotechnology, Shanghai, China). Afterward 20–30 μg protein of each group was subjected to polyacrylamide gel electrophoresis, and the protein bands were transferred to the Polyvinylidene difluoride (PVDF) membrane. The membrane was blocked with 5% milk at room temperature for 1 hour, followed by the incubation with the corresponding primary antibodies (Abcam, Cambridge, UK) overnight at 4°C: anti-*DOK7* (ab75049, 1:1000), anti-GAPDH (ab8245, 1:1000); anti-JAK1 (ab133666, 1:1000), anti-p-JAK1 (ab138005, 1:1000), anti-JAK2 (ab108596, 1:1000), anti-p-JAK2 (ab32101, 1:1000), anti-JAK3 (ab203611, 1:1000) and anti-p-JAK3 (ab61102, 1:1000). After washing the membrane was further probed with HRP-linked secondary antibody (1:3000; #7074 Cell Signaling, MA, USA) for 1 hour. The signal development was conducted using an enhanced chemiluminescence kit (Santa Cruz, TX, USA) and the protein bands were visualized on a gel imager system (Bio-Rad, CA, USA) [24].

### 5-ethynyl-2′-deoxyuridine (EdU) staining assay

We performed EdU staining assay using the BeyoClick™ EdU Cell Proliferation Kit (Beyotime, Beijing, China) and carried out the assay in the relevant experimental groups according to the instructions of the kit. The EdU staining signal in the nuclei was detected by Leica AM6000 microscope (Leica, Wetzlar, Germany) at 100X magnification.

### Cell cycle detection by flow cytometry

The DNA content (cell cycle) detection kit (Solarbio, Beijing, China) was used to analyze cell cycle distribution according to manufacturer’s instructions. Briefly, BLCA cells were resuspended in staining buffer at a concentration of 1 × 10^6^ cells/mL. The cells were fixed in cold 70% ethanol at −20 degree for 2 hours. Afterward the cells were stained in the buffer containing 100 μL RNase A solution and 400 μL Propidium Iodide (PI) at 4°C for 30 min. The DNA contents were determined by the BD FACS Canto™ II Flow Cytometer.

### Luciferase reporter assay

The *DOK7* wild-type luciferase reporter plasmid or the *DOK7* mutant luciferase reporter plasmid was co-transfected into HEK293 cells with miR-299-5p/miR-NC. 48 hours after the transfection, the relative luciferase activities were measured by the Fire-Lumi™ Luciferase Assay Kit (Nanjing GenScript Biotechnology Co., Ltd., Nanjing, China) according to the instructions.

### Cell count kit-8 (CCK-8) proliferation assay

BLCA cells with *DOK7* overexpression or knockdown were seeded in to a 96-well plate at 2000 cells/well and cultured for different duration. Accordingly, 10 μL CCK8 reagent (Solarbio, Beijing, China) was added to the cell culture for 3 hours in the cell culture incubator. The absorbance value (OD value) in each well was measured at 450 nm wavelength on a Synergy H1 microplate reader (VT, USA).

### Transwell chamber assay

BLCA cells were trypsinized and re-suspended in serum-free medium. The Transwell upper chamber (Corning, NY, USA) with the 8 μm pore size was used as the physical barrier for the migration assay, while the Transwell upper chamber coated with Matrigel (BD Biosciences, MA, USA) to mimic the extracellular matrix during tissue invasion was used for the invasion assay [29]. The cells in the serum-free medium were inoculated in the upper chamber at a cell density of 5 × 10^5^ cells/ml. The lower chamber was filled with 600 μl of complete medium. After 24 hours, the cells on the Transwell membrane were fixed and stained with 0.1% crystal violet for 30 min. After washing, the migrating or invading cells on the membrane were observed under a light microscope. For quantification, 5 random fields of each sample were counted at 100X magnification using Leica AM6000 microscope (Leica, Wetzlar, Germany).

### Immunohistochemical (IHC) staining

Tumor samples were harvested from the mice and fixed in 4% PFA for 12 h. The staining was performed in 5-μm sections of formalin-fixed paraffin-embedded (FFPE) tumor tissues. After deparaffinization and hydration, antigen retrieval was conducted by heating the sections in citrate unmasking solution at 95°C. The peroxidase activity in the sections were then inactivated by 3% hydrogen peroxide for 10 min. After washing three times in Tris-buffered saline with 0.1% Tween^®^ 20 detergent (TBST) buffer, the sections were blocked for 1 h at room temperature with 5% normal goat serum. The primary antibodies were used for staining in the sections overnight: anti-Ki-67 antibody (ab15580, 1:500, Abcam, Cambridge, UK) and anti-*DOK7* (ab75049, Abcam, Cambridge, UK). After the washes using TBST buffer, the section was further labeled in the SignalStain^®^ Boost Detection Reagent (HRP, Rabbit #8114, CST, MA, USA) for 30 min at room temperature before the imaging under the Leica AM6000 microscope (Leica, Wetzlar, Germany) at 200X magnification.

### Statistical analysis

Data presented in this study are expressed as the mean ± standard deviation of three independent measurements. The statistical difference between two groups was compared using unpaired student’s *t* test. Comparisons among multiple groups were analyzed using one-way analysis of variance (ANOVA) with Tukey’s post-hoc test for pairwise comparison. Comparisons of data at multiple time points were examined using two-way ANOVA. Univariate and multivariate Cox regression analysis was conducted to assess the overall survival in BLCA patients. Spearman correlation analysis was applied to determine the association between *DOK7* and miR-299-5p expression levels. Data were considered statistically significant when the *P* value was < 0.05.

### Availability of data and materials

The datasets used and/or analyzed during the current study are available from the corresponding author via email request.

## RESULTS

### *DOK7* expression level is a clinically relevant to the progression of BLCA

**Figure 1:**
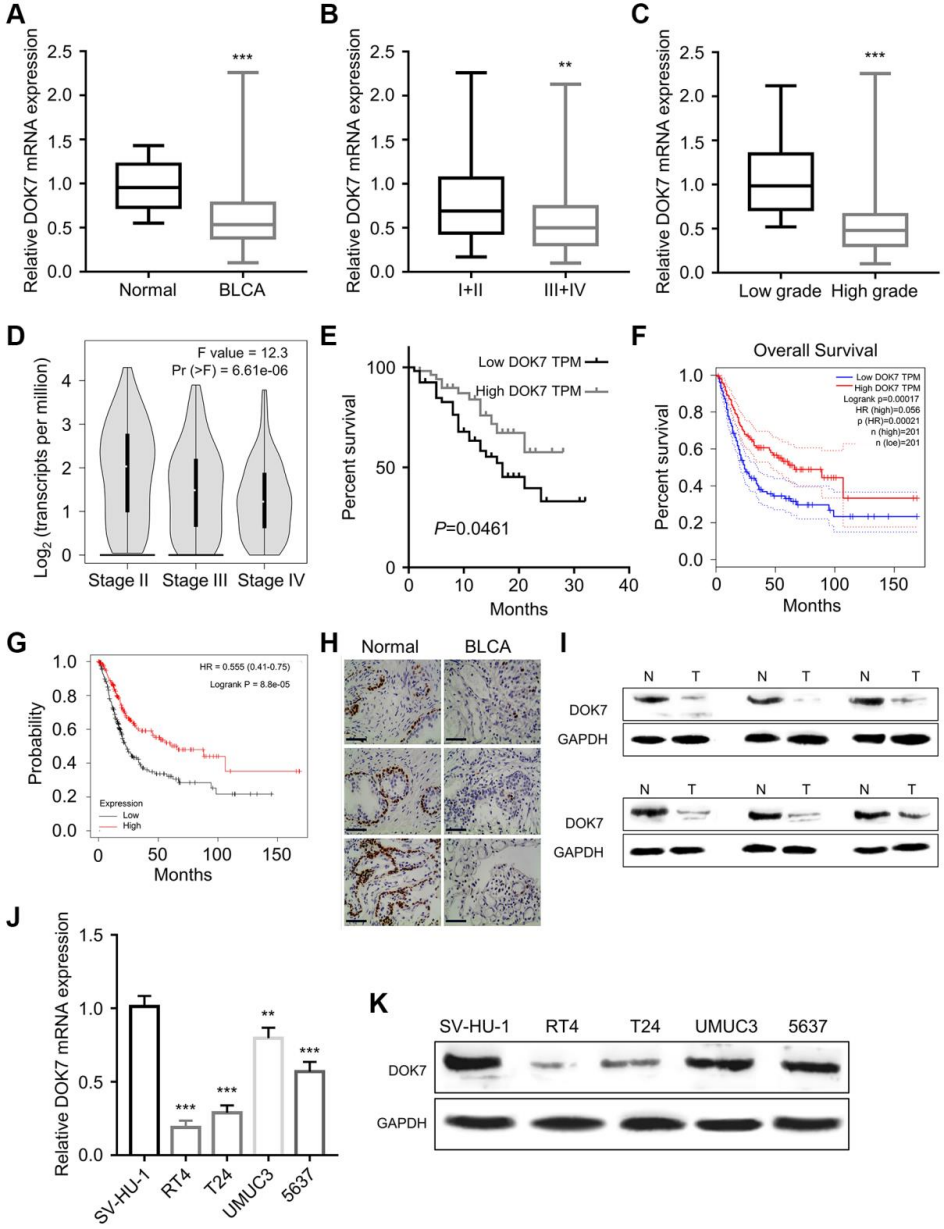
***DOK7* expression is suppressed in BLCA tumor and cell lines.** (**A**) The mRNA expression levels of *DOK7* in 108 pairs of BLCA cancer tissues (BLCA) and corresponding adjacent tissues (Normal) were detected by RT-qPCR method. *DOK7* expression was significantly decreased in BLCA*, P <* 0.01; (**B**) The mRNA expression levels of *DOK7* in BLCA tumor samples of different clinical stages (I+II and III+IV) were detected by RT-qPCR. *DOK7* levels were significantly decreased in III+IV stage, *P <* 0.01; (**C**) The expression levels of *DOK7* in BLCA tumor samples of different histological grades (Low and High) were detected by qRT-PCR. *DOK7* levels were significantly decreased in high grade tissues, *P <* 0.001; (**D**) The expression levels of *DOK7* in different clinical stages of TCGA-BLCA cancer tissues were analyzed by GEPIA. *DOK7* levels were gradually decreased with the advancement of the clinical stage, *P <* 0.001; (**E**) Kaplan–Meier (KM) curve analysis of the overall survival BLCA patients in *DOK7* low-expression group (*n* = 54) and high-expression group (*n* = 54). The overall survival rate of the low-expression group was significantly lower than that of the high-expression group, *P <* 0.001; (**F**) GEPIA online analysis of the TCGA BLCA cohort revealed the poor overall survival in BLCA patients with the low expression of *DOK7*; (**G**) K-M plotter online analysis found that the overall survival of BLCA patients with low expression of *DOK7* was poor; (**H**) IHC staining of *DOK7* protein expression levels in 3 pairs of cancer tissues and adjacent normal tissues under the magnification of 200X. *DOK7* expression was significantly reduced in BLCA tumor tissues. The *DOK7* staining was seen as dark signal in the nuclei, scale bar: 200 μm; (**I**) The protein levels of *DOK7* in 6 pairs of BLCA cancer tissues and normal tissues were detected by WB. *DOK7* protein levels were decreased in BLCA cancer tissues; (**J**) *DOK7* mRNA expression levels in BLCA cell lines (RT4, T24, and UMUC3, 5637) and normal bladder cell line (SV-HU-1) were detected by RT-qPCR; (**K**) WB detection of *DOK7* protein expression levels in BLCA cell lines and normal bladder cell line. ^**^P < 0.01; ^***^P < 0.001.

To investigate the role of *DOK7* in BLCA progression, we examined 108 pairs of BLCA tumors and para-cancerous normal specimens from BLCA patients. RT-qPCR results showed that *DOK7* mRNA expression levels in the para-cancerous tissues were significantly higher in comparison to BLCA tumor tissues (Figure 1A). In addition, we examined the *DOK7* expression levels in BLCA tissues of different clinical stages (I+II and III+IV) and different histological grades (Low and High). *DOK7* expression was significantly down-regulated in BLCA tumor tissues of more advanced clinical stages (Figure 1B, 1C). The expression pattern of *DOK7* in BLCA patients from the TCGA database was consistent with the observation in our clinical samples, showing that *DOK7* expression level gradually decreased with the advancement of the clinical stage (Figure 1D). Furthermore, Kaplan–Meier (KM) survival analyses using the 108 patients in our study (Figure 1E), TCGA BLCA dataset from GEPIA online tool (Figure 1F) and KM plotter online analysis (Figure 1G) revealed that a reduced *DOK7* expression level was associated with a poorer prognosis in BCLA patients.

In addition, we found that the expression level of *DOK7* was correlated with the histological grade and clinical stage of the tumor in the BLCA patients (*P* < 0.001), but there was no significant association with the patient’s age and gender, lymph node metastasis, distant metastasis, and tumor depth (Table 1). Through univariate COX regression analysis, we found that *DOK7* expression level, tumor depth, lymph node metastasis, distant metastasis, and the clinical stages showed statistically difference (*P* < 0.05) (Table 2). The multivariate COX regression analysis demonstrated that *DOK7* expression levels and the clinical stages in the BLCA patients were statistically significant (*P* < 0.05) (Table 2).

**Table 1:** Correlation between DOK7 expression and clinicopathological features.

**Parameters**	***N***	**DOK7 expression**		***P***
		**Low**	**High**	
**Age (years)**				
≤60	33	19	14	0.1934
>60	75	33	42	
**Gender**				
Male	72	41	31	0.1341
Female	36	15	21	
**Tumor depth**				
T0–T2	28	12	16	0.0883
T3–T4	73	45	28	
**Histological grade**				
Low	16	7	9	0.0407^*^
High	87	61	26	
**Clinical stage**				
I+II	32	11	21	0.0062^**^
III+IV	71	45	26	
**Lymph node metastasis**				
N0	63	27	36	0.1279
N1–N3	32	19	13	
**Distant metastasis**				
M0	86	38	48	0.1438
M1	12	8	4	

^*^p < 0.05, and ^**^p < 0.01.

**Table 2:** Univariate and multivariate Cox regression analysis of overall survival in bladder cancer patients.

**Variable**	**Univariate**			**Multivariate**		
	**HR**	**95% CI**	***P***	**HR**	**95% CI**	***P***
Age (>60/≤60)	1.261	0.628–1.738	0.241			
Gender (Female/Male)	1.418	0.785–1.637	0.617			
Grade (High/Low)	3.758	1.317–10.548	0.282			
Tumor depth (T3–T4/T0–T2)	2.421	1.582–3.747	0.002^**^	3.152	1.449–7.853	0.121
Clinical stage (III+IV/I+II)	2.229	1.426–3.439	<0.001^***^	0.522	0.287–0.953	0.03^*^
Lymph node (N1–N3/N0)	2.629	1.924–3.681	<0.001^***^	2.312	1.185–5.212	0.319
Distant metastasis (M1/M0)	3.728	1.271–6.685	<0.001^***^	1.259	0.859–3.429	0.592
DOK7 (High/Low)	1.855	1.412–2.752	<0.001^***^	2.416	1.323–4.317	0.003^**^

^*^p < 0.05, ^**^p < 0.01, and ^***^p < 0.001.

In the clinical samples, IHC staining and Western blot results revealed that the protein levels of *DOK7* in para-cancerous normal tissues were significantly higher compared to the tumor tissues (Figure 1H, 1I). The mRNA and protein levels of *DOK7* in BLCA cell lines (RT4, T24, and UMUC3, 5637) were also significantly lower when compared to the normal bladder cell line (SV-HU-1) (Figure 1J, 1K). Taken together, these findings suggest that *DOK7* down-regulation in BLCA might be relevant to the malignant progression.

### *DOK7* knockdown inhibits the proliferation and mobility in BLCA cells

**Figure 2:**
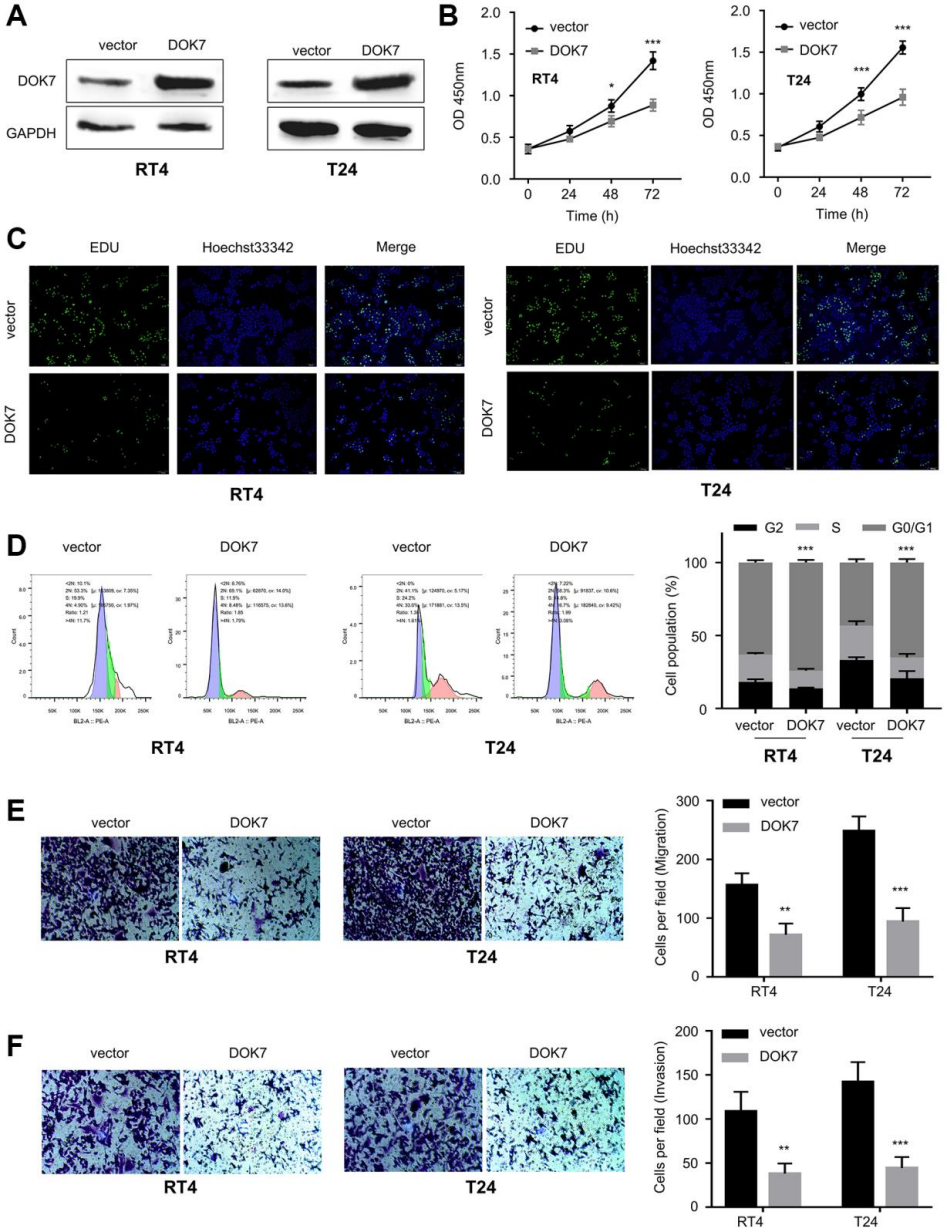
**Overexpression of *DOK7* inhibits the proliferation and invasion of BLCA cells.** (**A**) *DOK7* protein levels in the T24 and RT4 cells with or without *DOK7* overexpression detected by WB; (**B**) Cell proliferation of T24 and RT4 cells with or without *DOK7* overexpression was examined by CCK-8 assay at 0, 24, 48, 72 hours. *DOK7* overexpression suppressed cell proliferation; (**C**) EdU staining in assay T24 and RT4 cells with or without *DOK7* overexpression. Blue: nucleus; Red: EdU staining. Reduced EdU staining signal was observed in the *DOK7* overexpression group; (**D**) Cell cycle detection of different groups (vector and *DOK7*) of T24 and RT4 cells by flow cytometry. *DOK7* overexpression caused the cell cycle arrest at G1/S transition; (**E**, **F**) Transwell migration (**E**) and invasion (**F**) assays in T24 and RT4 cells with or without *DOK7* overexpression. 5 random fields of each sample were counted at 100X magnification. Scale bar: 50 μm. Reduced migration and invasion were observed in the *DOK7* overexpression group. ^**^P < 0.01; ^***^P < 0.001.

We next attempted to explore the biological function of *DOK7* in BLCA cells. BLCA cell lines (T24 and RT4, with a relatively low *DOK7* expression as shown in Figure 1K) were transduced with lentivirus carrying empty vector or *DOK7* overexpression sequences (Figure 2A). CCK8 proliferation assay and EdU staining assay revealed that the proliferation of BLCA cells was inhibited upon *DOK7* overexpression (Figure 2B, 2C). In addition, cell cycle analysis indicates that *DOK7* overexpression induced the G1/S arrest in BLCA cells (Figure 2D). Besides, Transwell migration and invasion assay further showed that *DOK7* overexpression significantly suppressed the migratory and invasive abilities of BLCA cells (Figure 2E, 2F).

**Figure 3:**
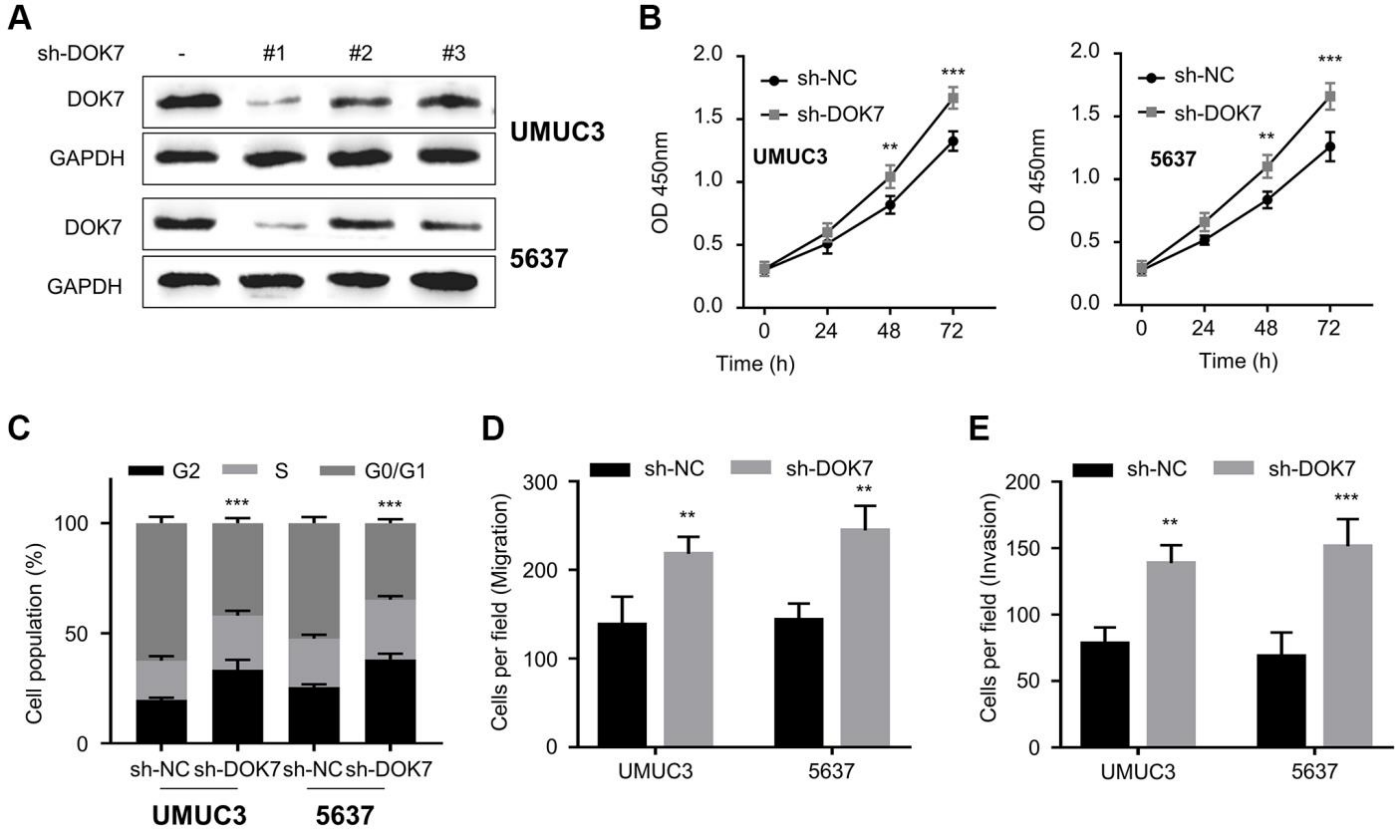
**Knockdown of *DOK7* promotes the proliferation and invasion of BLCA cells.** (**A**) Validation of knockdown efficiency of *DOK7* in UMUC3 and 5637 cell lines after the infection with lentivirus of sh-NC and sh-*DOK7* (#1-3) by RT-qPCR. sh-*DOK7* #1 showed the strongest silencing effect; (**B**) Cell proliferation of UMUC3 and 5637 cells with or without *DOK7* knockdown was examined by CCK-8 assay at 0, 24, 48, 72 hours. *DOK7* silencing promoted cell proliferation; (**C**) Cell cycle detection in different groups (sh-NC and sh-*DOK7*) of UMUC3 and 5637 cells by flow cytometry. *DOK7* silencing promoted cell proliferation promoted cell cycle progression; (**D**, **E**) Transwell migration (**D**) and invasion (**E**) assays in UMUC3 and 5637 with or without *DOK7* knockdown. 5 random fields of each sample were counted at 100X magnification. Scale bar: 50 μm. Enhanced migration and invasion were observed in the *DOK7* silencing group. ^**^P < 0.01; ^***^P < 0.001.

We also applied sh-RNA targeting *DOK7* to knock down *DOK7* in BLCA cells (UMUC3 and 5637 cell lines with a relatively high level of *DOK7* expression) (Figure 3A). sh-RNA#1 showed the strongest silencing effect in both cell lines, which was used for the subsequent experiments. Functional assays demonstrated that *DOK7* knockdown promoted the cell growth (Figure 3B) and cell cycle progression (Figure 3C), and enhanced the migration (Figure 3D) and invasion abilities (Figure 3E) in BLCA cells. Collectively, our data suggest that *DOK7* serves as a tumor suppressor to impair the aggressive phenotype in BLCA cells.

### miR-299-5p/*DOK7* axis suppresses the malignancy of BLCA cells

**Figure 4:**
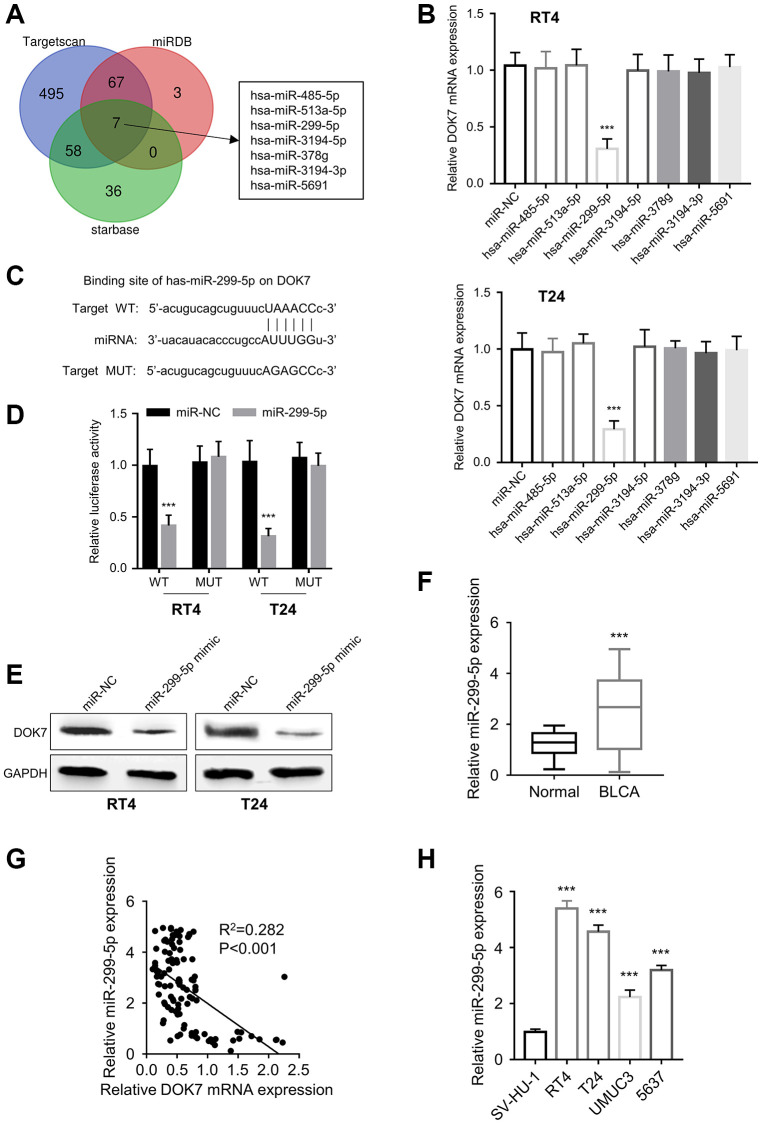
**miR-299-5p targets *DOK7*.** (**A**) *DOK7*-targeting miRNA candidates predicted by “starbase”, “Targetscan” and “miRDB” databases; (**B**) RT-qPCR analysis of *DOK7* mRNA levels after the transfection of miR-NC or the miRNA mimics of hsa-miR-485-5p, hsa-miR-513a-5p, hsa-miR-299-5p, hsa-miR-3194-5p, hsa-miR-378g, hsa-miR-3194-3p and hsa-miR-5691; (**C**) The predicted binding site of miR-299-5p and *DOK7* mRNA 3'UTR region by “TargetScan”; (**D**) Luciferase reporter assay to detect the binding ability of miR-299-5p to wild-type *DOK7* and mutant *DOK7* luciferase reporter; (**E**) WB detection of *DOK7* protein levels in different groups (miR-NC, miR-299-5p mimic) of T24 and RT4 cell lines; (**F**) RT-qPCR detection of miR-299-5p expression in 108 pairs of BLCA cancer tissues and adjacent normal tissues; (**G**) Spearman correlation coefficient analysis of the relationship between *DOK7* and miR-299-5p expression levels in BLCA tumor tissues; (**H**) RT-qPCR detection of miR-299-5p expression levels in BLCA cell lines and normal bladder cell line. ^***^P < 0.001.

We next sought to explore the underlying molecular mechanism by which *DOK7* becomes down-regulated in BLCA cells. Using “Starbase”, “Targetscan” and “miRDB” databases, a total of seven common *DOK7*-targeting miRNAs were predicted: hsa-miR-485-5p, hsa-miR-513a-5p, hsa-miR-299-5p, hsa-miR-3194-5p, hsa-miR-378g, hsa-miR-3194-3p, hsa-miR-5691 (Figure 4A). Upon the transfection of miR-NC and corresponding miRNA mimics in T24 and RT4 cells, only miR-299-5p mimic was able to down-regulate the mRNA level of *DOK7* (Figure 4B). To verify the binding between miR-299-5p and the 3’ untranslated region of *DOK7* mRNA, we constructed the luciferase reporter containing wild type (WT) binding sites or the mutated (MUT) binding sites (Figure 4C). Luciferase reporter assay results showed that compared with miR-NC, miR-299-5p mimic was able to suppress the activity of WT reporter, while there was no inhibition observed in the MUT reporter (Figure 4D). miR-299-5p overexpression by miRNA mimic caused the reduction of *DOK7* protein expression in BCLA cells (Figure 4E). In the clinical samples, miR-299-5p expression was up-regulated in BLCA cancer tissues when compared to the para-cancerous ones (Figure 4F). Spearman correlation coefficient analysis revealed a significant negative correlation between *DOK7* expression and miR-299-5p levels in BLCA tumor tissues (Figure 4G). In addition, the expression levels of miR-299-5p in BLCA cell lines were also up-regulated when compared to the normal bladder cell line (Figure 4H). These data suggest that *DOK7* is a downstream target of miR-299-5p in BLCA cells.

**Figure 5:**
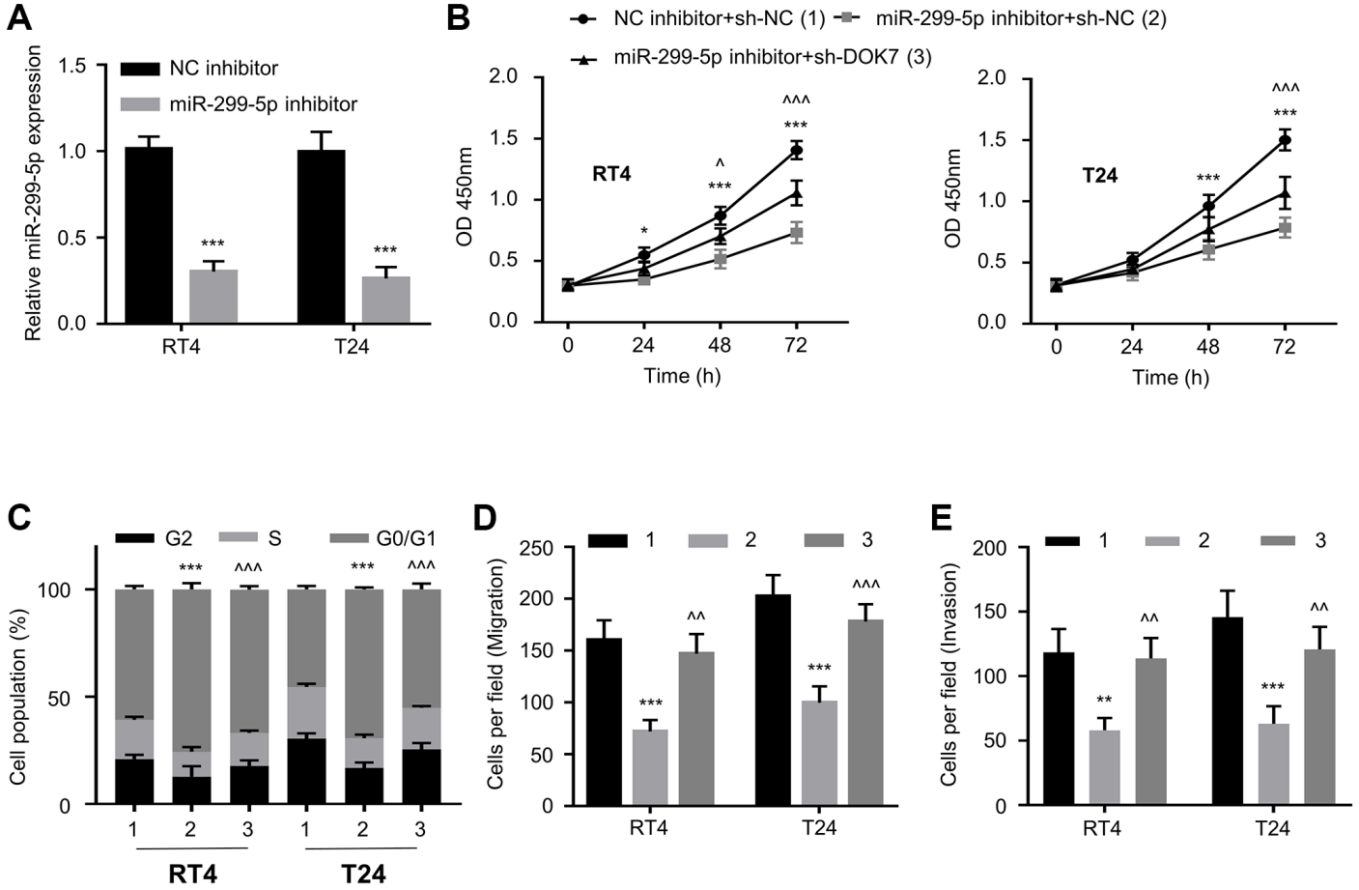
**Knockdown of *DOK7* reverses the effect of miR-299-5p inhibitor on BLCA cells.** (**A**) RT-qPCR detection of miR-299-5p levels in different groups (NC inhibitor, miR-299-5p inhibitor) of T24 and RT4 cells; (**B**) Cell proliferation in different groups (NC inhibitor + sh-NC, miR-299-5p inhibitor + sh-NC, or miR-299-5p inhibitor+sh-*DOK7*) of T24 and RT4 cells was examined by CCK-8 assay at 0, 24, 48, 72 hours; (**C**) Cell cycle detection in different groups of T24 and RT4 cells by flow cytometry; (**D**, **E**) Transwell migration (**D**) and invasion (**E**) assays in different groups of T24 and RT4 cells. 5 random fields of each sample were counted at 100X magnification, scale bar: 50 μm. ^**^P < 0.01; ^***^P < 0.001.

We next wonder whether miR-299-5p impacts on *DOK7*-dependent regulatory effect on BLCA cells. To test this hypothesis, we employed an inhibitor of miR-299-5p to suppress the expression level of miR-299-5p in BCLA cells (Figure 5A). CCK8 assay showed that miR-299-5p inhibitor significantly suppressed the proliferation of BLCA cells; while the knockdown of *DOK7* partially restored the cell growth (Figure 5B). In addition, cell cycle analysis revealed that *DOK7* knockdown also relieved the G1/S transition arrest caused by miR-299-5p inhibition (Figure 5C). Furthermore, miR-299-5p inhibitor significantly impaired the migration and invasion in BLCA cells, and *DOK7* knockdown abrogated the inhibitory effects (Figure 5D, 5E). Therefore, miR-299-5p/*DOK7* axis regulates the malignant phenotype of BLCA cells.

### *DOK7* negatively regulates JAK/STAT signaling pathway in BLCA cells

**Figure 6:**
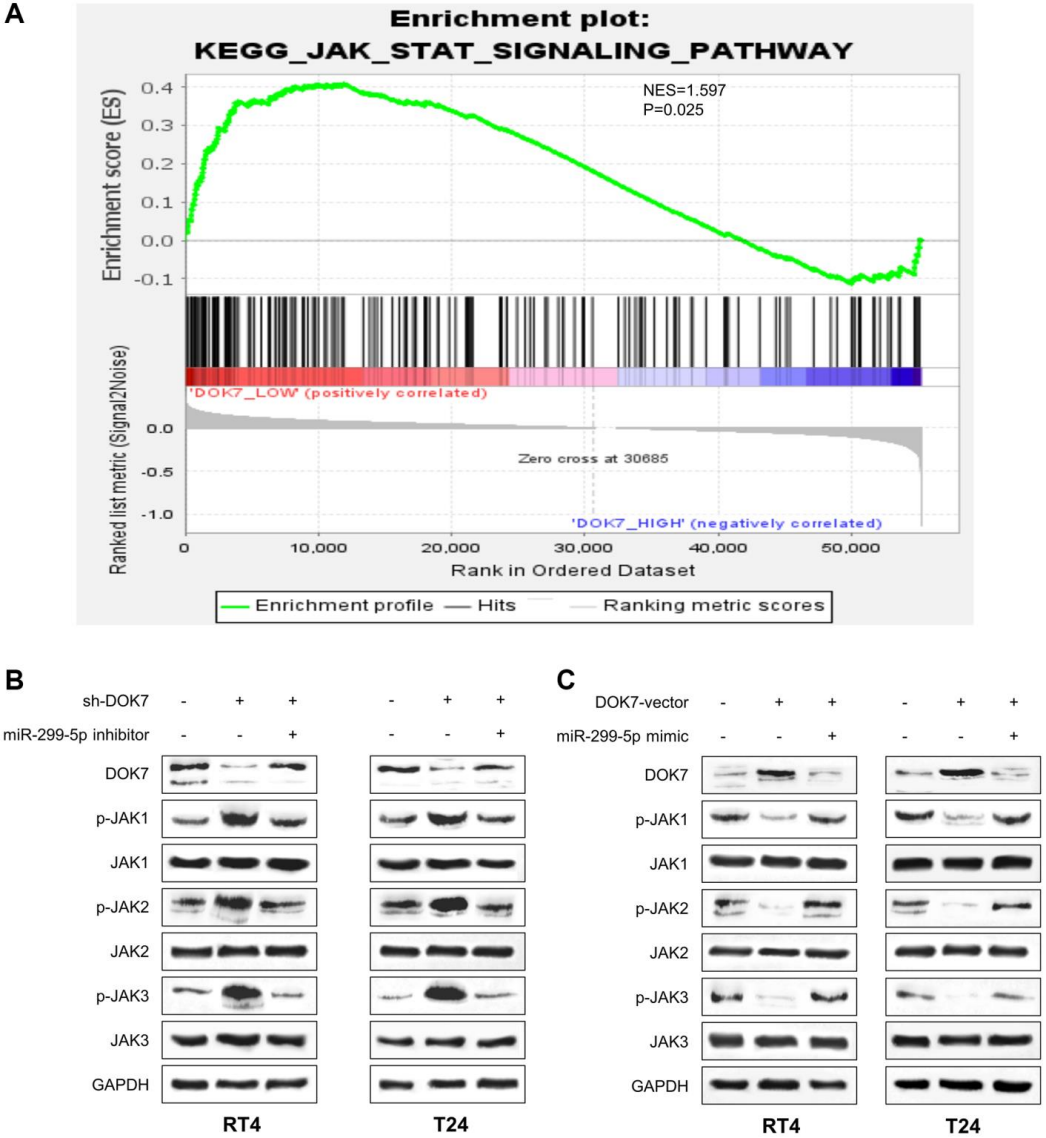
***DOK7* negatively regulates JAK/STAT3 pathway in BLCA cells.** (**A**) GESA analysis of TCGA BLCA dataset revealed that the low expression of *DOK7* is associated with JAK/STAT signaling pathway activation; (**B**) WB detection of *DOK7* protein level and phosphorylation levels of JAK1-3 as indicated in different groups (sh-NC+NC inhibitor, sh-*DOK7*+NC inhibitor and sh-*DOK7*+miR-299-5p inhibitor) of T24 and RT4 cells. (**C**) WB detection of *DOK7* protein level and phosphorylation levels of JAK1-3 as indicated in different groups (vector+miR-NC, *DOK7*-vector+miR-NC and *DOK7*-vector+miR-299-5p mimic) of T24 and RT4 cells.

Next, we performed gene set enrichment analysis (GSEA) using the BLCA cohort data in the TCGA database, which revealed that the low expression of *DOK7* was associated with the significant enrichment of JAK/STAT3 pathway gene expression (Figure 6A). To validate the observation, we performed Western blot to examine the phosphorylation of JAK1, JAK2 and JAK3 upon *DOK7* overexpression or knockdown. Upon *DOK7* silencing the levels of p-JAK1, p-JAK2 and p-JAK3 were elevated in T24 and RT4 cells, which was could be suppressed by the transfection of miR-299-5p inhibitor (Figure 6B). On the contrary, *DOK7* overexpression reduced the levels of p-JAK1, p-JAK2 and p-JAK3, and this effect was abolished by miR-299-5p mimic (Figure 6C). Thus, these data suggested that *DOK7* is a negative regulator of JAK/STAT signaling pathway in BLCA cells.

### *DOK7* overexpression inhibits the tumor formation of BLCA cells *in vivo*

**Figure 7:**
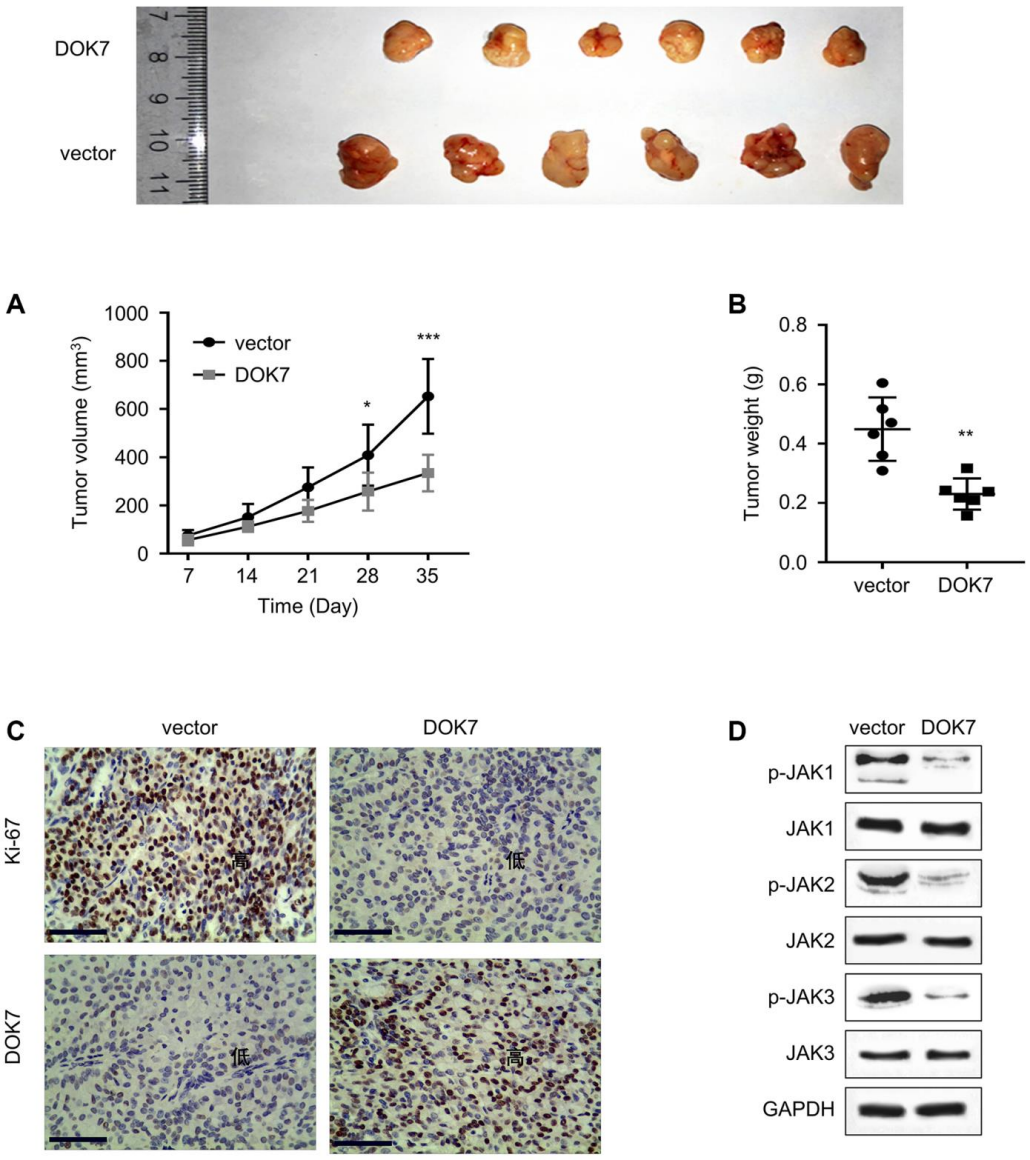
***DOK7* overexpression suppresses the tumor formation of BLCA cells *in vivo.*** (**A**) Tumor growth was measured every week after the inoculation of T24 cells carrying empty vector or *DOK7* expression vector (*n* = 6 mice in each group); (**B**) The summary of tumor weight in different groups (vector and *DOK7*) after mouse sacrifice on day 35; (**C**) IHC staining of *DOK7* and Ki-67 protein expression levels in different groups of tumor samples (vector and *DOK7*). Both *DOK7* and Ki67 were stained as the dark signal in the nuclei, scale bar: 200 μm; (**D**) WB detection of the phosphorylation levels of JAK1-3 in different groups of tumor samples (vector and *DOK7*). ^*^P < 0.05; ^**^P < 0.01; ^***^P < 0.001.

To further evaluate the tumor suppressive function of *DOK7 in vivo*, T24 cells stably expressing the control empty vector after lentivirus infection (vector group) or T24 cells stably expressing *DOK7* after lentivirus infection (*DOK7* group) were inoculated into nude mice subcutaneously. The monitoring of the tumor growth and tumor weight showed that *DOK7* overexpression significantly suppressed the tumorigenesis of T24 cells in nude mice (Figure 7A, 7B). IHC staining of the tumor tissues showed that the high level of *DOK7* expression significantly reduced the levels of the proliferating maker Ki-67 (Figure 7C). In addition, *DOK7* overexpression also reduced the levels of p-JAK1, p-JAK2 and p-JAK3 in the tumor tissues (Figure 7D). Together, these data support that *DOK7* serves as a tumor suppressor in BLCA possibly by targeting JAK signaling pathway.

## DISCUSSION

In this present study, we identified *DOK7* as a potential tumor suppressor in BLCA. *DOK7* was significantly down-regulated in BLCA tissue and cell lines, and its low expression level was associated with the dismal prognosis in BLCA patients. *DOK7* overexpression suppressed the cell proliferation and tumorigenesis of BLCA cells. On the contrary, silencing *DOK7* promoted the aggressiveness of BLCA cells. We further showed that *DOK7* serves as a negative regulator of JAK signaling pathway in BLCA cells.

In agreement with our data, *DOK7* was reported as a tumor suppressor in glioma, breast and lung cancer [19, 20, 30–33]. For example, *DOK7* was found to be down-regulated in lung cancer and its reduced expression was associated with the poor survival in lung cancer patients [30–32]. In breast cancer, *DOK7* suppresses the cell growth and mobility by targeting PI3K/AKT pathway [20]. In glioma, *DOK7* expression was repressed by DNMT1 and *DOK7* down-regulation facilitates the proliferation of glioma cells [19]. Together, our data and previous studies support the notion that targeting *DOK7* could be a strategy to mitigate the aggressiveness of cancer cells in different malignancies.

We further identified miR-299-5p as the negative regulator of *DOK7*. miR-299-5p was significantly up-regulated in BLCA tumor and cell lines. miR-299-5p overexpression reduced *DOK7* protein levels in BLCA cells. Besides, miR-299-5p inhibition could suppress the aggressiveness in BLCA cells. These data indicate that miR-299-5p functions as an oncogenic factor to negatively regulate *DOK7* in BLCA. miRNAs are short non-coding RNAs which negatively target the downstream mRNA target [11]. The deregulation of different miRNAs has been implicated in the progression of BLCA [12–16]. In previous studies, there were also evidence that miR-299-5p promotes the cell growth in different tumors. For instance, miR-299-5p facilitates the G1/S progression and enhances cell proliferation acute promyelocytic leukemia [34]. Similar results were also observed in osteosarcoma that miR-299-5p promotes cell cycle transition to accelerate cell growth [35]. However, there is also data indicating the tumor suppressor role of miR-299-5p in other types of cancers. miR-299-5p was reported to suppress cell metastasis in breast cancer by negatively regulating serine/threonine kinase 39 [36]. Therefore, the role of miR-299-5p in different cancers may be divergent due to the different downstream targets.

We further showed that *DOK7* is a negative regulator of JAK signaling pathway in BLCA cells. *DOK7* overexpression suppressed the phosphorylation of JAK1-3, while *DOK7* silencing showed the opposite effect. JAK/STAT signaling is aberrantly activated in a wide spectrum of tumors to promote the malignant progression of cancer [37]. In normal scenarios, JAK/STAT pathway functions to induce the production of different growth factors, cytokines and hormones to regulate cellular physiology [38]. However, the constitutive activation of this pathway was found to promote the aggressiveness in breast and esophageal cancer, and the elevated phosphorylation levels of JAK1 and STAT3 were linked with the dismal prognosis [39, 40]. In line with these findings, our data suggest that *DOK7* dampens the activation of JAK/STAT signaling to suppress the aggressiveness of BLCA cells. However, the mechanism by which *DOK7* negatively regulates the activity of JAK/STAT signaling pathway is unclear.

There are several limitations regarding the findings of our data. First, the mechanism by which miR-299-5p becomes up-regulated in BLCA tumor and cells remains elusive. The understanding of the regulatory mechanisms of miR-299-5p expression could provide insights into targeting *DOK7*. Second, the role of miR-299-5p in regulating the tumorigenesis of BLCA cells needs to be further validated in the animal model. Further, the mechanism by which *DOK7* negatively regulates the JAK/STAT signaling pathway warrants future investigation.

In conclusion, our study uncovered the role of miR-299-5p/*DOK7* axis in dictating the malignancy of BLCA cells. *DOK7* serves as a tumor suppressor which was down-regulated in BLCA tumor and cells. The reduced level of *DOK7* seems to be regulated by the increased level of miR-299-5p. Targeting miR-299-5p/*DOK7* axis could mitigate the aggressiveness of BLCA cells. This study reveals novel molecular mechanism related to the progression of BLCA, and provides new therapeutic targets for BLCA management.
